# Frequency of Silent Myocardial Ischemia in Diabetic Patients Presenting With Non-cardiac Complaints

**DOI:** 10.7759/cureus.89722

**Published:** 2025-08-10

**Authors:** Lakhveer Rathi, Taha Hassan Habib, Mohammad Ibrahim Rasool, Orji Victor Ifunanya, Javeria Binte Khalid Jamil, Sheheryar Khan, Muhammad Usama Saud Bin Nasir, Aqib Latif, Fazal Ghaffar, Hafiz Ali Raza

**Affiliations:** 1 Rheumatology, University Hospital Limerick, Limerick, IRL; 2 Medicine, Niazi Medical and Dental College, Sargodha, PAK; 3 Pediatrics, Naseer Teaching Hospital/Kabir Medical College, Peshawar, PAK; 4 Medicine, Lagos State University Teaching Hospital, Lagos, NGA; 5 Community Medicine, Darulsehat Hospital/LCMD (Liaquat College of Medicine and Dentistry), Karachi, PAK; 6 Medicine, District Head Quarter Teaching Hospital DIKhan/Gomal Medical College (GMC), Peshawar, PAK; 7 Emergency Medicine, Barnet Hospital Royal Free London NHS Trust, London, GBR; 8 Internal Medicine, Lady Reading Hospital, Peshawar, PAK; 9 Cardiology, King Fahad Military Medical Complex, Dhahran Kingdom of Saudi Arabia, Riyadh, SAU; 10 Agriculture Extension, Muhammad Nawaz Shareef University of Agriculture, Multan, PAK

**Keywords:** cvd, diabetes treatment, non compliance, patients satisfaction, smi

## Abstract

Background: Silent myocardial ischemia (SMI) is a common but frequently overlooked manifestation of coronary artery disease in patients with diabetes mellitus, largely due to autonomic neuropathy and atypical symptom presentation.

Objective: To determine the frequency of SMI in diabetic patients presenting with non-cardiac complaints using electrocardiography (ECG), serum troponin-I, and non-invasive stress testing, and to assess its association with clinical risk factors.

Methods: This cross-sectional observational study was conducted at Niazi Medical and Dental College, Niazi Welfare Foundation Teaching Hospital, from June 2023 to June 2024. A total of 255 diabetic patients were enrolled through consecutive non-probability sampling. Data were collected on demographics, duration of diabetes, associated comorbid conditions (e.g., hypertension, dyslipidemia), and current medications. Investigations included a resting 12-lead ECG, random blood glucose, serum troponin-I, HbA1c, and lipid profile.

Results: The mean age of participants was 56.8 ± 10.9 years, and 56.5% were male. Ischemic changes on ECG were noted in 43 patients (16.9%), and 12 patients (4.7%) had elevated troponin-I levels. Stress testing was performed in 55 patients, confirming SMI in 39 (15.3%). SMI was significantly associated with a duration of diabetes >10 years (p = 0.02), HbA1c >8% (p < 0.01), and hypertension (p = 0.04). No significant association was found with age (p = 0.09) or gender (p = 0.19).

Conclusion: SMI is prevalent in diabetic patients presenting with non-cardiac complaints, with poor glycemic control and long-standing diabetes being strong predictors. Routine cardiac screening should be considered in high-risk asymptomatic diabetics to enable early detection and timely intervention.

## Introduction

Cardiovascular disease (CVD) remains the predominant cause of death and disability among individuals with diabetes mellitus (DM), with coronary artery disease (CAD) forming a major component of this burden. Silent myocardial ischemia (SMI), defined as objective findings of myocardial ischemia in the absence of chest pain or usual anginal symptoms, is an insidious but clinically important form of CAD [1]. SMI is prevalent worldwide in diabetic populations, and it is usually unrecognized because of unusual or absent symptoms until it leads to major adverse cardiac events (MACE), which could be acute myocardial infarction, heart failure, or sudden cardiac death [2]. The absence of symptomatic warning signs in SMI qualifies it as a diagnostic problem and a silent killer that greatly impairs the process of managing cardiovascular risk in diabetic patients [3].

There are several processes behind the heightened incidence of SMI in diabetics. A typical complication of longstanding diabetes is autonomic neuropathy, which leads to the dulling of pain transmission in the myocardium and thus conceals the characteristic ischemic pain. Moreover, atherosclerosis is enhanced by endothelial dysfunction, augmented arterial stiffness, and low-grade chronic inflammation in diabetic individuals, contributing to an elevated risk of myocardial ischemia even in non-stressful conditions [4]. All these pathophysiological alterations not only complicate the diagnosis of ischemia but also predispose diabetics to more complicated coronary lesions at the time of presentation. The clinical significance of SMI is represented by a close correlation with poor outcomes [5]. It has been amply proven in the literature that diabetic patients with unrecognized ischemia carry similar and, in many cases, worse prognoses than those with symptomatic CAD. In addition, there are indications that SMI could be a substantial cause of sudden cardiac deaths among them [6]. Despite these dangers, SMI is an under-researched condition, particularly among patients with non-cardiac complaints-a frequent situation in outpatient and emergency care. Such non-cardiac appearances can be in the form of infections, metabolic derangements, abdominal discomfort, dizziness, or generalized fatigue, and are therefore frequently given priority over cardiovascular evaluation, leading to missed chances of timely diagnosis [7].

Diagnosis of SMI commonly depends on non-invasive tests, including resting ECG, exercise treadmill testing, myocardial perfusion scintigraphy, or stress echocardiography. Silent ischemia, in fact, in certain instances may even be incidentally detected on routine preoperative studies or imaging done asymptomatically to investigate another problem [8]. The lack of symptoms, however, means that clinicians are unlikely to actively request such tests unless there is a high index of suspicion [9]. This highlights the importance of improved clinical awareness and risk-based screening approaches, especially in high-risk subgroups, including older diabetics, individuals with numerous comorbidities, or those with abnormal ECGs despite non-specific complaints [10]. The international guidelines have long given ambivalent advice about routine screening of asymptomatic diabetic patients for CAD because of issues related to cost-effectiveness and prognostic value [11]. Nevertheless, selective screening founded on clinical characteristics and the presentation scenario, such as screening during non-cardiac complaint visits, could provide a trade-off [12]. The early identification of SMI in these environments allows clinicians to implement preventive measures such as antiplatelet therapy, statin use, glycemic control optimization, and lifestyle counseling that may prevent a future cardiovascular event. In developing states such as Pakistan, where the healthcare setup is frequently overwhelmed and the prevalence of diabetes is increasing exponentially, the detection of SMI via pragmatic, opportunistic screening can result in a revolutionary change in cardiovascular outcomes [13].

Objective

To determine the frequency of SMI in diabetic patients presenting with non-cardiac complaints and to assess its association with clinical risk factors.

## Materials and methods

This was a cross-sectional, observational study conducted at the Department of Medicine, Niazi Medical and Dental College and its affiliated Niazi Welfare Foundation Teaching Hospital, Sargodha, Pakistan. The study period spanned 12 months, from June 2023 to June 2024. The institution serves as a tertiary care referral center, catering to a diverse urban and rural population, providing a representative sample of diabetic patients in the region. A consecutive non-probability sampling technique was employed to recruit eligible participants. The sample size was calculated based on an expected prevalence of SMI of 20%, a 95% confidence level, a 5% margin of error, and a 10% expected attrition rate, resulting in a minimum required sample of 240 patients. To ensure adequate power and account for potential exclusions, 255 patients were ultimately enrolled. Patients aged 18 years or older with a confirmed diagnosis of type 2 diabetes mellitus according to the American Diabetes Association (ADA) diagnostic criteria, presenting to the hospital with non-cardiac primary complaints, were included in the study. Only patients who voluntarily provided written informed consent were included. Exclusion criteria were applied to eliminate potential confounders and included a known history of ischemic heart disease or prior myocardial infarction, presentation with chest pain or typical anginal symptoms, evidence of established infarction on baseline ECG, end-stage chronic kidney disease requiring dialysis, severely deranged serum electrolytes at admission, pregnancy, or incomplete clinical or laboratory records.

Data collection

All enrolled participants underwent a structured clinical evaluation performed by trained physicians. This included detailed history-taking, focusing on the duration of diabetes, treatment adherence, and the presence of comorbid conditions such as hypertension, dyslipidemia, or obesity. A thorough physical examination was conducted to document vital signs, body mass index (BMI), and cardiovascular status. Laboratory investigations included random blood glucose, glycated hemoglobin (HbA1c), fasting lipid profile, serum creatinine, serum electrolytes, and serum troponin-I. All patients underwent a standard resting 12-lead electrocardiogram (ECG). Patients with either abnormal ECG findings indicative of ischemia or elevated serum troponin-I in the absence of cardiac symptoms were further evaluated for SMI using a stress test. Stress testing was performed either by treadmill exercise ECG (Bruce protocol) or stress echocardiography, based on the patient’s ability to exercise and institutional resources. SMI was diagnosed if inducible ischemia was confirmed on stress testing in asymptomatic patients. SMI was operationally defined in this study as the presence of inducible ischemia confirmed on stress testing (treadmill ECG or stress echocardiography) in asymptomatic patients who initially showed either abnormal ECG findings or biochemical markers such as troponin-I without typical cardiac symptoms. Hypertension was defined as systolic blood pressure ≥140 mmHg or diastolic blood pressure ≥90 mmHg, or current use of antihypertensive medication. Dyslipidemia was defined as total cholesterol ≥200 mg/dL, LDL ≥130 mg/dL, HDL <40 mg/dL in men or <50 mg/dL in women, triglycerides ≥150 mg/dL, or use of lipid-lowering therapy. Data were recorded on predesigned proformas and double-checked by two independent investigators to minimize data entry errors. Laboratory testing and ECG interpretation were standardized and supervised by senior consultants to ensure consistency. Missing or incomplete data were cross-verified from medical records whenever possible.

Data analysis

All collected data were coded and entered into IBM SPSS Statistics for Windows, Version 26 (Released 2019; IBM Corp., Armonk, New York) for analysis. Continuous variables such as age, duration of diabetes, HbA1c, and lipid levels were summarized using means and standard deviations, while categorical variables such as gender, presence of hypertension, dyslipidemia, and SMI were expressed as frequencies and percentages. Bivariate associations between SMI and categorical clinical factors were evaluated using chi-square or Fisher’s exact tests, as appropriate. Continuous variables were compared using independent-samples t-tests or Mann-Whitney U tests, depending on the normality of distribution. To assess the independent predictors of SMI, multivariate logistic regression analysis was performed, reporting adjusted odds ratios (aOR) with 95% confidence intervals. A p-value of <0.05 was considered statistically significant.

## Results

A total of 255 diabetic patients were included in the study, with a mean age of 56.8 ± 10.9 years. Males comprised 56.5% (n = 144) of the sample, while females accounted for 43.5% (n = 111). The mean duration of diabetes was 8.2 ± 4.7 years, with hypertension present in 159 patients (62.4%) and dyslipidemia in 97 patients (38%). Electrocardiographic findings revealed ischemic changes in 43 patients (16.9%), while 12 patients (4.7%) had mildly elevated troponin-I levels. Based on these initial findings, 55 patients (21.6%) underwent further non-invasive stress testing (Table 1).

**Table 1:** Demographic and Clinical Characteristics of the Study Population SMI: silent myocardial ischemia.

Variable	Value
Mean age (years)	56.8 ± 10.9
Gender - Male	144 (56.5%)
Gender - Female	111 (43.5%)
Mean duration of diabetes (years)	8.2 ± 4.7
Hypertension	159 (62.4%)
Dyslipidemia	97 (38.0%)
Frequency of silent myocardial ischemia (SMI)	
ECG with ischemic changes	43 (16.9%)
Mildly elevated troponin-I	12 (4.7%)
Underwent stress testing	55 (21.6%)
Confirmed SMI (via stress test in asymptomatic patients with initial ECG/troponin abnormalities)	39 (15.3%)
Overall frequency of SMI	39/255 (15.3%)

Among patients with SMI, a higher proportion were aged ≥60 years 18 (46.2%) compared to those without SMI 76 (35.2%), although this difference did not reach statistical significance (p = 0.09). A significantly greater percentage of patients with SMI had diabetes for more than 10 years (61.5% vs. 28.7%, p = 0.02) and poor glycemic control, as indicated by HbA1c >8% (76.9% vs. 31.9%, p < 0.01). Hypertension was also more prevalent in the SMI group 29 (74.4%) than in the non-SMI group 130 (60.2%), with statistical significance (p = 0.04). While males made up a larger share of the SMI group 25 (64.1%) compared to the non-SMI group 119 (55.1%), this difference was not statistically significant (p = 0.19) (Table 2).

**Table 2:** Association of Clinical Factors With Silent Myocardial Ischemia Chi-square test (χ²) was applied for categorical variables to assess association between clinical factors and SMI. SMI: silent myocardial ischemia, HbA1c: glycated hemoglobin.

Factor	SMI Present (n = 39)	SMI Absent (n = 216)	p-value	χ² value
Age ≥60 years	18 (46.2%)	76 (35.2%)	0.09	2.87
Duration of diabetes >10 years	24 (61.5%)	62 (28.7%)	0.02	5.47
HbA1c >8%	30 (76.9%)	69 (31.9%)	<0.01	19.21
Hypertension	29 (74.4%)	130 (60.2%)	0.04	4.21
Gender—Male	25 (64.1%)	119 (55.1%)	0.19	1.70

Variables that were statistically significant in the bivariate analysis (p<0.05) were entered into a multivariate logistic regression model to identify independent predictors of SMI. The adjusted odds ratios (AOR) and 95% confidence intervals are presented in Table 3.

**Table 3:** Multivariate Logistic Regression to Identify Independent Predictors of SMI. SMI: silent myocardial ischemia; HbA1c: glycated hemoglobin; aOR: adjusted odds ratio; CI: confidence interval.

Factor	Adjusted Odds Ratio (aOR)	95% Confidence Interval (CI)	p-value
HbA1c >8%	4.28	2.02 – 9.07	<0.01
Duration of diabetes >10 years	2.56	1.17 – 5.60	0.02
Hypertension	2.09	1.01 – 4.35	0.04
Age ≥60 years	1.48	0.71 – 3.08	0.29
Male Gender	1.23	0.59 – 2.58	0.58

The highest frequency of SMI was observed among patients presenting with general fatigue or malaise, with 22.6% (7 out of 31) affected. Gastrointestinal complaints and infections also showed notable SMI rates of 17.2% (11 out of 64) and 16.9% (12 out of 71), respectively. Musculoskeletal pain and neurological symptoms were associated with lower frequencies of SMI at 11.6% (5 out of 43) and 15.4% (4 out of 26). No cases of SMI were identified among patients presenting with dermatological or other miscellaneous issues (Table 4).

**Table 4:** Distribution of Silent Myocardial Ischemia by Type of Non-cardiac Complaint SMI: silent myocardial ischemia; HbA1c: glycated hemoglobin.

Type of Complaint	Total (n)	SMI Present (n)	SMI Frequency (%)
Infections (UTI, RTI, etc.)	71	12	16.9%
Gastrointestinal complaints	64	11	17.2%
Musculoskeletal pain	43	5	11.6%
General fatigue/malaise	31	7	22.6%
Neurological complaints	26	4	15.4%
Others (e.g., dermatological)	20	0	0.0%
Total	255	39	15.3%

Patients in the SMI group had significantly higher mean HbA1c levels (9.1 ± 1.3%) compared to those without SMI (7.6 ± 1.1%), indicating poorer glycemic control (p < 0.01). Additionally, the mean duration of diabetes was longer in the SMI group (10.2 ± 3.5 years) than in the non-SMI group (7.7 ± 4.2 years), and this difference was also statistically significant (p = 0.01) (Table 5).

**Table 5:** Comparison of Mean HbA1c and Diabetes Duration in SMI vs Non-SMI Groups Independent-samples t-test was applied for continuous variables comparing mean HbA1c and diabetes duration between SMI and non-SMI groups. SMI: silent myocardial ischemia; HbA1c: glycated hemoglobin.

Variable	SMI Group (n=39)	Non-SMI Group (n=216)	p-value	t-value
Mean HbA1c (%)	9.1 ± 1.3	7.6 ± 1.1	<0.01	7.12
Mean duration of diabetes (years)	10.2 ± 3.5	7.7 ± 4.2	0.01	3.44

## Discussion

This study revealed a notable frequency of SMI in diabetic patients presenting with non-cardiac complaints, with 15.3% of the total cohort showing objective evidence of ischemia in the absence of anginal symptoms. According to previous research, SMI is more common in diabetics because of diabetic autonomic neuropathy, which reduces the perception of cardiac pain. In our study, ischemic changes on ECG were present in 16.9% of the cohort, and subsequent stress testing confirmed SMI in nearly three-fourths of those tested. These results highlight the potential role of routine or targeted cardiovascular assessment in asymptomatic individuals and the limited utility of symptom-based screening in diabetic populations [13]. The prevalence of SMI was higher in patients with more prolonged diabetes and elevated HbA1c. The SMI patients were found to have a longer duration of diabetes (mean 10.2 ± 3.5 years) than non-SMI patients (7.7 ± 4.2 years), and their glycemic control was significantly poorer (HbA1c 9.1% vs. 7.6%, p < 0.01). These results are in line with the postulation that cumulative glycemic load plays a role in endothelial dysfunction, microvascular ischemia, and accelerated atherosclerosis, which in turn predispose patients to SMI. Thus, the duration of diabetes and inadequate glycemic control might become significant clinical predictors of early cardiovascular screening [14].

In addition, hypertension showed a significant correlation with SMI (p = 0.04), which confirms the fact that systemic hypertension is well known to be associated with an elevated risk of coronary artery disease. In our sample, age and being male were not statistically significant predictors; nevertheless, the tendency for the prevalence of SMI to be higher in older people and in males is consistent with the epidemiology of coronary artery disease worldwide [15]. Evaluation of non-cardiac presenting complaints showed that the relative frequency of SMI was higher among the patients presenting with general fatigue or infections and gastrointestinal symptoms. Silent ischemia was present in 22.6% of the patients who presented with fatigue. The clinical implications of this observation cannot be underestimated because non-specific signs such as fatigue are usually ignored during a standard cardiovascular assessment, especially in low-resource environments [16].

These findings prompt the necessity of opportunistic screening approaches, particularly in individuals with long-standing diabetes, suboptimal glycemic control, and comorbid hypertension, even when they seek health care for other unrelated health issues. Non-invasive stress testing, serum troponin assays, and resting ECG can be potentially available screening tools that are inexpensive and widely accessible to reveal subclinical cardiac disease [17]. As the worldwide prevalence of diabetes and cardiovascular morbidity rises, active detection of SMI may play an important role in decreasing the occurrence of major adverse cardiac events [18].

Limitations

This study has several limitations. First, it was conducted at a single tertiary care center, which may limit the generalizability of the findings to broader populations. Second, the diagnostic approach relied on the availability and feasibility of stress testing, which may have excluded some patients unable to undergo testing. Third, more advanced cardiac imaging modalities, such as myocardial perfusion scintigraphy or coronary CT angiography, were not utilized due to resource constraints. Additionally, other potential risk factors for SMI, such as family history of ischemic heart disease and physical activity levels, were not assessed. Finally, as follow-up was not performed, the long-term prognostic implications of SMI in this cohort remain unknown.

Future directions

Future multi-center longitudinal studies are recommended to evaluate the prognostic impact of early SMI detection and targeted interventions in diabetic patients, including the role of additional risk factors and more advanced imaging tools.

## Conclusions

It is concluded that SMI is a significant and under-recognized cardiovascular complication in diabetic patients, particularly those presenting with non-cardiac complaints. The study found that 39 (15.3%) of such patients had objective evidence of myocardial ischemia despite the absence of typical symptoms. Long duration of diabetes, poor glycemic control, and coexisting hypertension were identified as important predictors of SMI. These findings highlight the importance of opportunistic cardiac screening in asymptomatic diabetic individuals, especially those with multiple risk factors, to enable early detection and intervention.
